# Fibroblasts as a practical alternative to mesenchymal stem cells

**DOI:** 10.1186/s12967-018-1536-1

**Published:** 2018-07-27

**Authors:** Thomas E. Ichim, Pete O’Heeron, Santosh Kesari

**Affiliations:** 1 SpinalCyte LLC, Houston, TX USA; 2 Department of Translational Neurosciences and Neurotherapeutics, John Wayne Cancer Institute and Pacific Neuroscience Institute, Santa Monica, CA USA

**Keywords:** Stem cell therapy, Regenerative medicine, Disc degenerative disease, Fibroblasts, Mesenchymal stem cells

## Abstract

Mesenchymal stem cell (MSC) therapy offers great potential for treatment of disease through the multifunctional and responsive ability of these cells. In numerous contexts, MSC have been shown to reduce inflammation, modulate immune responses, and provide trophic factor support for regeneration. While the most commonly used MSC source, the bone marrow provides relatively little starting material for cellular expansion, and requires invasive extraction means, fibroblasts are easily harvested in large numbers from various biological wastes. Additionally, in vitro expansion of fibroblasts is significantly easier given the robustness of these cells in tissue culture and shorter doubling time compared to typical MSC. In this paper we put forward the concept that in some cases, fibroblasts may be utilized as a more practical, and potentially more effective cell therapy than mesenchymal stem cells. Anti-inflammatory, immune modulatory, and regenerative properties of fibroblasts will be discussed in the context of regenerative medicine.

## Introduction

Friedenstein and colleagues were the first to describe mesenchymal stem cells (MSC) as adherent cells derived from bone marrow that were capable of forming colonies and comprising the radioresistant fraction of cells associated with hematopoiesis [1, 2]. There cells are currently defined as adherent, non-hematopoietic cells expressing markers such as CD90, CD105, and CD73, while lacking expression of CD14, CD34, and CD45, and being able to differentiate into adipocytes, chondrocytes, and osteocytes in vitro after treatment with differentiation inducing agents [3]. Although early studies in the late 1960s initially identified MSC in the bone marrow [4], more recent studies have reported these cells can be purified from various tissues such as adipose [5], heart [6], Wharton’s Jelly [7], dental pulp [8], peripheral blood [9], cord blood [10], and more recently menstrual blood [11–13]. Studies in the bone marrow showed that although MSC are the primary cell type that overgrows in in vitro cultures, in vivo MSC are found at a low ratio compared to other bone marrow mononuclear cells, specifically, 1:10,000 to 1:100,000 [14]. The physiological role of MSC still remains to be fully elucidated, with one hypothesis being that bone marrow MSC act as precursors for stromal cells that make up the hematopoietic stem cell microenvironment [15–17].

The first clinical use of MSC was reported in a 1995 paper, in which Lazarus et al. reported the use of autologous, in vitro expanded, “mesenchymal progenitor cells” to treat 15 patients suffering from hematological malignancies in remission. The authors demonstrated that a 10 cc bone marrow sample was capable of 16,000-fold growth over a 4–7 week in vitro culture. Cell administration was performed in total doses ranging from 1 to 50 × 10^6^ cells and was not causative of treatment associated adverse effects [18]. In a subsequent study from the same group, the use of MSC to accelerate hematopoietic reconstitution was performed in 28 breast cancer patients who received high dose chemotherapy. MSC at concentrations of 1.0–2.2 × 10^6^/kg, were administered intravenously with no treatment associated adverse effects. The authors noted that leukocytic and thrombocytic reconstitution occurred at an accelerated rate as compared to historical controls [19]. It is important to note that these initial clinical experiences with MSC were in patients with oncological indications and no overt acceleration of cancer progression was noted. This has been a concern given that MSC are known to be angiogenic [20–25], produce mitogenic/antiapoptotic factors [26–32], and exert an immune suppressive effect [33–40]. Besides feasibility, these studies were important because they established the technique for ex vivo expansion and re-administration.

The demonstration of clinical feasibility, combined with animal models supporting therapeutic efficacy of MSC in non-hematopoietic indications [41–48], gave rise to a series of clinical trials with MSC in a wide range of therapeutic areas ranging from major diseases such as stroke [49–52], heart failure [53, 54], COPD [55], and liver failure [56], as well as rare diseases such as osteogenesis imperfecta [57], Hurler syndrome [58], and Duchenne Muscular Dystrophy [59]. The ability to generate large amounts of defined MSC starting with a small clinical sample, to administer without need for haplotype matching, and excellent safety profile, has resulted in a current 367 clinical trials listed on the international registry clinicaltrials.gov. While some trials have demonstrated efficacy of MSC, little is known about molecular mechanisms. Initial studies demonstrated ability of certain MSC types to differentiate into functional tissues that is compromised as a result of the underlying pathological.

Although MSC appear to be ideal as a source for development of cellular therapeutics, there are several drawbacks. Firstly, MSC are relatively rare cells in tissue, with bone marrow containing 1:10,000 to 1:100,000 MSC per nucleated cells [14]. In order to generate therapeutically relevant doses (1–2 million/kg), the MSC need to undergo massive numbers of cell multiplication in vitro. This increases both the possibility of mutatgenesis, as well as loss of activity. Accordingly, tissue sources, such as fibroblasts, in which larger numbers of cells may be originally extracted, may serve as an attractive alternative to MSC. Secondly, fibroblasts typically possess a shorter doubling time than MSC, allowing for less tissue culture media use in their expansion, thus reducing cost of production. Finally, MSC generation often involves isolation and in vitro growth of the cellular product. In contrast, fibroblasts may be grown without need for isolation of specific subtypes of cells.

## Properties of fibroblasts

Fibroblasts comprise the main cell type of connective tissue, possessing a spindle-shaped morphology, whose classical function has historically been believed to produce extracellular matrix responsible for maintaining structural integrity of tissue. Fibroblasts also play an important role in proliferative phase of wound healing, resulting in deposition of extracellular matrix [60, 61]. During wound healing, scar tissue is formed by fibroblast over proliferation. In embryos, and in some types of amphibians, scarless healing occurs after injury, by processes which are currently under intense investigation [62, 63]. With aging, many kinds of tissues and organs undergo fibrosis gradually, such as fibrosis of skin, lung, liver, kidney and heart. The process of scar tissue formation is caused by hyperproliferation of fibroblasts, as well as these cells producing abnormally large amounts of extracellular matrix and collagens during proliferation and thereby replacing normal organ structure (parenchyma), leading to functional impairment and scar formation, which may further trigger persistent fibrosis.

The original thinking on fibroblasts was that these cells possess similar characteristics regardless of their source of origin, a notion that is no longer believed to be entirely correct [64]. For example, studies have shown that protein antigens such as MHC II [65], C1q receptor [66], LR8 [67], and Thy-1 [68], differ in expression based on tissue origin of fibroblasts. Interestingly, not only origin of fibroblasts affects markers but also proliferating state. For example, one study showed that CD40 expression on fibroblasts was elevated on proliferating fibroblasts but reduced on non-proliferating cells [69]. Other variations in fibroblasts have been detected in various tissues for example, lung fibroblasts are known to possess variable expression of both cell surface marker expression, as well as in their levels of collagen production [70]. Fibroblasts derived from periodontal tissue possess differences in extracellular matrix production, glycogen pools, and morphology [71].

At present there is a deficiency in specific and reproducible markers for fibroblasts, which has hampered to some extent, our knowledge of in vivo functionality [72, 73]. Currently one of the main means of detecting fibroblasts is quantification of vimentin expression. Vimentin, is major structural component of the intermediate filaments in many cell types, is shown to play an important role in vital mechanical and biological functions such as cell contractility, migration, stiffness, stiffening, and proliferation [74, 75]. One disadvantage of this marker is that it is also found on endothelial cells of capillaries that often locate very close to fibroblasts, additionally, it is found on neurons [76]. Another marker of fibroblasts, that is preferentially found on cardiac derived fibroblasts is the collagen receptor Discoidin Domain Receptor 2 (DDR2 [77]). Unfortunately DDR2 has also been found non-fibroblast cells such as neutrophils [78], dendritic cells [79, 80], and osteoblasts [81]. Investigators typically refer to Thy-1, also known as CD90, as a marker associated with fibroblasts. CD90 is comprised of a glycoprotein anchored by a glycosylphosphatidylinositol (GPI) tail [82], which is found at various levels of expression of fibroblasts [70]. Expression of CD90 on fibroblasts has been detected on cells from mouse and rat lungs, as well as from reproductive tract and ocular tissues [68, 83–85]. There appears to be correlations between expression of CD90 and particular function of fibroblasts. For example, in one study, lung fibroblasts where shown to be heterogenous in expression of CD90. Cells expressing CD90 where more susceptible to apoptosis than cells lacking its expression. Furthermore it was found that CD90 co-aggregated with Fas, a death inducing molecule, on lipid rafts, allowing for increased ability for apoptosis [86]. In other studies, CD90 expression is related to ability to produce the inflammatory cytokine IL-6 [87].

## Mesenchymal properties of fibroblasts: differentiation

The therapeutic significance of the ability of MSC to differentiate into various tissues is under debate, with some arguments being that MSC exert disease inhibiting activity by secretion of soluble factors, while other schools of thought suggest that MSC actually differentiate into injured tissue. The possibility that fibroblasts can also differentiate into tissue is intriguing. Classically, the role of the fibroblast has been perceived to strictly allow for collagen and other ECM deposition, as well as formation of scar tissue.

The association of fibroblasts with differentiation potential comes originally from studies showing that when mechanical stimulation is applied to dermal fibroblast cells encapsulated in alginate beads using a custom-built bioreactor system for either a 1- or 3-week period at a frequency of 1 Hz for 4 h/day under hypoxic conditions, chondrogenic differentiation of the fibroblasts was observed, as indicated by elevated aggrecan gene expression and an increased collagen production rate [88]. In vivo ability of fibroblasts to differentiate into chondrogenic cells was demonstrated in a subsequent study. The group of Professor An from Rush University induced disc degeneration in New Zealand white rabbits by annular puncture and after 4 weeks intradiscally implanted human dermal fibroblasts or saline. Eight weeks after cellular implantation there was a significant increase in disc height in the treated compared to control fibroblasts, as well as reduced expression of inflammatory markers, a higher ratio of collagen type II over collagen type I gene expression, and more intense immunohistochemical staining for both collagen types I and II [89]. A subsequent study by an independent group where 8 rabbits underwent disc puncture to induce disc degeneration. One month later, cultured fibroblasts, which had been taken from the skin, were injected into the disc. The viability and the potential of the injected cells for reproduction were studied histologically and radiologically. Cellular formations and organizations indicating to the histological recovery were observed at the discs to which fibroblasts were transplanted. The histological findings of the discs to which no fibroblasts were transplanted, did not show any histological recovery. Radiologically, no finding of the improvement was found in both groups. The fibroblasts injected to the degenerated discs are viable [90].

In addition to differentiation into chondrocytic tissues, other studies have shown that fibroblasts are capable of differentiating into other types of cells. In one study, cultured human adult bronchial fibroblast-like cells (Br) where assessed in comparison with mesenchymal cell progenitors isolated from fetal lung (ICIG7) and adult bone marrow (BM212) tissues. Surface immunophenotyping by flow cytometry revealed a similar expression pattern of antigens characteristic of marrow-derived MSCs, including CD34 (−), CD45 (−), CD90/Thy-1 (+), CD73/SH3, SH4 (+), CD105/SH2 (+) and CD166/ALCAM (+) in Br, ICIG7 and BM212 cells. There was one exception, STRO-1 antigen, which was only weakly expressed in Br cells. Analysis of cytoskeleton and matrix composition by immunostaining showed that lung and marrow-derived cells homogeneously expressed vimentin and nestin proteins in intermediate filaments while they were all devoid of epithelial cytokeratins. Additionally, alpha-smooth muscle actin was also present in microfilaments of a low number of cells. All cell types predominantly produced collagen and fibronectin extracellular matrix as evidenced by staining with the monoclonal antibodies to collagen prolyl 4-hydroxylase and fibronectin isoforms containing the extradomain (ED)-A together with ED-B in ICIG7 cells. Br cells similarly to fetal lung and marrow fibroblasts were able to differentiate along the three adipogenic, osteogenic and chondrogenic mesenchymal pathways when cultured under appropriate inducible conditions. Altogether, these data indicate that MSCs are present in human adult lung. They may be actively involved in lung tissue repair under physiological and pathological circumstances [91].

Another study revealing multilineage differentiation of fibroblasts used cells isolated from juvenile foreskins. These cells where shown to share a mesenchymal stem cell phenotype and multi-lineage differentiation potential. Specifically, the investigators demonstrated similar expression patterns for CD14(−), CD29(+), CD31(−), CD34(−), CD44(+), CD45(−), CD71(+), CD73/SH3–SH4(+), CD90/Thy-1(+), CD105/SH2(+), CD133(−) and CD166/ALCAM(+) in well-established adipose tissue derived-stem cells and foreskin fibroblastic cells by flow cytometry. Immunostainings showed that fibroblast cells expressed vimentin, fibronectin and collagen; they were less positive for alpha-smooth muscle actin and nestin, while they were negative for epithelial cytokeratins. When cultured under appropriate inducible conditions, both cell types could differentiate along the adipogenic and osteogenic lineages. Additionally, fibroblasts demonstrated a higher proliferation potential than mesenchymal stem cells. These findings are of particular importance, because skin or adipose tissues are easily accessible for cell transplantations in regenerative medicine [92]. Verification of multilineage differentiation of foreskin fibroblasts was provided by a study in which foreskin fibroblasts where demonstrated to possess shorter doubling time than MSC, as well as ability to multiply more than 50 doublings without undergoing senescence. The cells were positive for the MSC markers CD90, CD105, CD166, CD73, SH3, and SH4, and could be induced to differentiate into osteocytes, adipocytes, neural cells, smooth muscle cells, Schwann-like cells, and hepatocyte-like cells [93].

Other more detailed studies have evaluated the potential of fibroblasts to differentiate into endodermal cell lineages. For example, in one publication, fibroblast cells were isolated from 12- to 14-day-old pregnant mice that were characterized for their surface markers and tri-lineage differentiation potential. The investigators found that islet-like cell aggregates (ICAs) were produced in some cultures, which was confirmed for their pancreatic properties via immunofluorecence for C-peptide, glucagon, and somatostain. They were positive for CD markers-Sca1, CD44, CD73, and CD90 and negative for hematopoietic markers-CD34 and CD45 at both transcription and translational levels. The transcriptional analysis of the ICAs at different day points exhibited up-regulation of islet markers (Insulin, PDX1, HNF3, Glucagon, and Somatostatin) and down-regulation of MSC-markers (Vimentin and Nestin). They positively stained for dithizone, C-peptide, insulin, glucagon, and somatostatin indicating intact insulin producing machinery. In vitro glucose stimulation assay revealed three-fold increase in insulin secretion as compared to basal glucose with insulin content being the same in both the conditions. In vivo data on ICA transplantation showed reversal of diabetes in streptozotocin induced diabetic mice. These results demonstrated that mouse fibroblast cells are capable of differentiation into insulin producing cell aggregates [94].

Another study using foreskin fibroblasts assessed whether these cells can be transdifferentiated into hepatocytes. The investigators demonstrated that when fibroblasts where cultured in distinct media, spheres formed in Dulbecco’s modified Eagle’s medium (DMEM) containing F12, epidermal growth factor (EGF), and basic fibroblast growth factor (b-FGF), however fibroblast-like morphology was attained with the cells where cultured in DMEM-based growth medium. Both cell populations expressed the typical mesenchymal stem cell markers CD90, CD105, and CD73, but the p75 neurotrophin receptor (p75NTR) was detected only in fibroblast derived spheres. Both types of fibroblasts could differentiate into hepatocyte-like cells, which express typical liver markers, including albumin and hepatocyte paraffin 1 (Hep Par1), along with liver-specific biological activities. When plasmids containing the human hepatitis B virus (HBV) genome were transfected transiently into fibroblasts, differentiated hepatocyte-like cells secrete large amounts of HBe and HBs antigens [95].

## Mesenchymal properties of fibroblasts: immune modulation

One of the major therapeutic properties of MSC is believed to be immune modulation. In fact, the original clinical implementation of MSC where not for treatment of degenerative diseases but for the immune mediated disorder called “graft versus host disease” (GVHD), which occurs subsequent to allogeneic hematopoietic transplants in which donor cells begin attacking recipient cells [96, 97]. MSC have also shown promise in other immunologically mediated conditions such as multiple sclerosis [98, 99], sepsis [100], type 1 diabetes [101], and rheumatoid arthritis [102]. Give that fibroblasts appear to share with MSC surface markers and ability to transdifferentiate into various tissues, it may not be unreasonable to assess whether fibroblasts possess immune modulatory properties such as MSC do.

One of the first investigations into immune modulatory activities of fibroblasts compared foreskin fibroblasts to bone marrow MSC. The investigators found that fibroblasts were unable to provoke in vitro allogeneic reactions, but strongly suppress lymphocyte proliferation induced by allogeneic mixed lymphocyte culture (MLC) or mitogens. We show that fibroblasts’ immunosuppressive capacity is independent from prostaglandin E2, IL-10 and the tryptophan catabolising enzyme indoleamine 2,3-dioxygenase and is not abrogated after the depletion of CD8+ T lymphocytes, NK cells and monocytes [103].

In another study, human foreskin fibroblasts (HFF) where assessed for immune modulatory potential. It was demonstrated that HFFs suppressed human peripheral blood mononuclear cells (PBMC) proliferation stimulated with mitogen or in an allogeneic mixed lymphocyte reaction comparable to BMSCs. However, HFFs showed undetectable levels of indoleamine 2,3-dioxygenase and inducible nitric oxide synthase expression, in contrast to BMSCs when cocultured with activated PBMCs. To identify HFF specific immunosuppressive factors, the investigators performed array profiling of common cytokines expressed by HFFs and BMSCs alone or when cocultured with activated PBMCs. Real-time polymerase chain reaction analysis confirmed that multiple factors were upregulated in HFFs cocultured with activated PBMCs compared with HFFs alone or BMSCs cultured under the same conditions. Functional assays identified interferon-α as the major immunosuppressive mediator expressed by HFFs. These results suggest that the HFFs possess immunosuppressive properties, which are mediated by alternate mechanisms to that reported for BMSCs [104].

A more rigorous study attempted to overcome possible various between different fibroblast cell lines, in order to assess whether the immune modulatory activity of the fibroblasts was a peculiarity to the cells used, or whether it was an overall property of the cell type itself. The investigators used four well-established human fibroblast strains from three different tissue sources and several human MSC strains from two different tissue sources to compare the phenotypic and immunological characteristics of these cells. The investigators found that fibroblast strains had a similar morphology to MSCs, expressed the same cell surface markers as MSCs and could also differentiate into adipocytes, chondrocytes and osteoblasts. Also, similar to MSCs, these fibroblasts were capable of suppressing T cell proliferation and modulating the immunophenotype of macrophages. They also showed that MSCs deposit extracellular matrices of collagen type I and fibronectin, and express FSP1 in patterns similar to fibroblasts. Based on currently accepted definitions for cultured human MSCs and fibroblasts, the investigators could not find any immunophenotypic property that could make a characteristic distinction between MSCs and fibroblasts [105].

## Clinical use of fibroblasts

Foreskin fibroblasts, together with keratinocytes have been used clinically for treatment of various non-healing wounds. One of the earliest studies created a cultured skin substitute by successive cultivation of fibroblasts and keratinocytes that were combined within a collagen matrix. This collagen matrix was composed of a collagen spongy sheet and a collagen gel. The collagen spongy sheet was designed to produce a honeycomb structure having many holes in which all holes through the sheet were filled with collagen gel. This specific structure thereby allows for the nourishment of the cultured keratinocytes on the surface of the matrix when placed on the graft bed. In this study, autologous cultured skin substitute was applied to a 51-year-old man who had sustained a burn injury. Three sheets of the cultured skin substitute (6 × 9.5 cm) were grafted onto the full-thickness excised wound in the right anterior chest wall. One week after grafting most of the matrix disappeared and stratified keratinocytes were seen to have firmly attached to the underlying tissue. Five weeks after grafting a cornified epidermal layer was seen. Ten months after grafting a mature epidermis and a well-differentiated papillary and reticular dermis replacement were observed. The physical properties and color of this grafted area resemble those of normal skin. In the second patient case, autologous cultured skin substitute was applied to a 30-year-old man with a scar remaining after tattoo removal. Eight sheets of the cultured skin substitute (10 × 18 cm) were applied on an excised wound (thickness, 0.02–0.025 in.) of both the fore- and upper arms. The histological appearance of a biopsied skin specimen from the grafted area at 3 months after grafting showed a mature epidermis and a well-differentiated reticular dermis replacement. The regenerated skin at 14 months after grafting showed an excellent result [106].

Numerous other studies have been conducted using a similar type of approach, which culminated in the commercial product known as Dermagraft. The FDA approved Dermagraft^®^ is a sterile, cryopreserved, human fibroblast-derived dermal substitute generated by the culture of neonatal dermal fibroblasts onto a bioabsorbable polyglactin mesh scaffold. During the product-manufacturing process, the human fibroblasts proliferate to fill the interstices of this scaffold and secrete collagen, other extracellular matrix proteins, growth factors, and cytokines, creating a three-dimensional human tissue containing metabolically active living cells. Dermagraft has been approved for marketing in the United States for the treatment of diabetic foot ulcers. In addition, the product is in active development for the treatment of venous leg ulcers and has been clinically used in a variety of other indications to stimulate wound healing [107].

Another example of allogeneic fibroblasts in clinical use is the treatment of the genetic disease recessive dystrophic epidermolysis bullosa, which is characterized by a mutation leading to reduced collagen VII production. Patients with this condition possess very fragile skin, which often blisters and sheds with minimal contact. A phase II double-blinded randomized controlled trial of intralesional allogeneic cultured fibroblasts in suspension solution versus suspension solution alone for wound healing in patients with recessive dystrophic epidermolysis bullosa was reported. Patients were screened for chronic ulcers and reduced collagen VII expression. Up to 6 pairs of symmetric wounds were measured and biopsied at baseline, then randomized to cultured allogeneic fibroblasts in a crystalloid suspension solution with 2% albumin or suspension solution alone. Ulcer size, collagen VII protein and messenger RNA expression, anchoring fibril numbers, morphology, and inflammatory markers were measured at 2 weeks and at 3, 6, and 12 months. The investigators reported that all wounds healed significantly more rapidly with fibroblasts and vehicle injections, with an area decrease of 50% by 12 weeks, compared with noninjected wounds. Collagen VII expression increased to a similar degree in both study arms in wounds from 3 of 5 patients [108]. Another study also supported this possible clinical application. Collagen VII deficient patients were erosions were randomized 1:1, to either a single treatment of 5 × 10(6) fibroblasts per linear cm of erosion margin or vehicle. All subjects continued standard wound care. Twenty-six erosions in 11 subjects with recessive dystrophic epidermolysis bullosa were injected; 14 erosions received fibroblasts and 12 vehicle alone. A single series of injections was given at day 0 and all follow-up visits were completed. Treatment difference between fibroblasts and vehicle was − 23.5% [95% confidence interval (CI) − 3.5 to − 43.5, P = 0.025] at day 7, − 19.15% (95% CI 3.36 to − 41.66, P = 0.089) at day 14 and − 28.83% (95% CI 7.97 to − 65.63, P = 0.11) at day 28 [109]. The ability of fibroblasts to replace collagen VII production indicates that it is feasible to clinically utilize allogeneic cells without need for immune suppression or development of rejection reactions. This is in line with previous studies that we discussed which suggested fibroblasts possess similar immunological properties to MSC, and thus could be used allogeneically.

Several other examples of clinical use of autologous fibroblasts exist. These include in conditions of gingival repair [110, 111], inhibition of wrinkles [112], and treatment of acne scars [113]. LAVIV^®^ (azficel-T) is a FDA approved autologous cellular product indicated for improvement of the appearance of moderate to severe nasolabial fold wrinkles in adults. This product involves administration of 18 million laboratory expanded fibroblasts and is currently in commercial use without an significant side effects associated with injection [114].

## Conclusion

Despite the great interest in development of MSC as an allogeneic cellular therapeutic, the commercially attractive, and medically beneficial properties of allogeneic fibroblasts have been overlooked, with exception of dermal regeneration. The authors believe that based on the literature overviewed, sufficient rational exists for expanding clinical investigations of fibroblast therapeutics in areas of unmet medical need. Currently, the company SpinalCyte, of which two of the authors are members of, is conducting a clinical trial in disc degenerative disease for which enrollment has been completed and interim data is pending. To the knowledge of the authors, this will be the first allogeneic use of fibroblasts outside of skin conditions. Success of these studies is likely to advance the clinical translation of fibroblasts into other areas of regenerative medicine.
